# Ablative laser surgery for allergic tattoo reactions: a retrospective study

**DOI:** 10.1007/s10103-020-03164-2

**Published:** 2020-10-26

**Authors:** S. A. S. van der Bent, Sanne Huisman, T. Rustemeyer, A. Wolkerstorfer

**Affiliations:** Academic Tattoo Clinic Amsterdam, Department of Dermatology, Amsterdam University Medical Centre, Location AMC, Meibergdreef 9, 2205 AZ Amsterdam, The Netherlands

**Keywords:** Red tattoo, Contact dermatitis, Ablative laser, CO_2_ laser, Patient-reported outcome

## Abstract

Patients with allergic tattoo reactions are burdened with itch and have a reduced quality of life. Conservative treatment is often insufficient and little is known about treatment options to remove the responsible allergen. We aimed to address the effectiveness and safety of ablative laser therapy including measurement of patient’s satisfaction, in patients with allergic reactions to tattoos. A retrospective study was conducted including patients with allergic tattoo reactions who were treated with a 10,600 nm ablative CO_2_ laser, either by full-surface ablation or fractional ablation. Clinical information originated from medical files and a 25-item questionnaire. Sixteen tattoo allergy patients were treated with a CO_2_ laser between January 2010 and January 2018. Fourteen patients completed the questionnaire. Ten patients were satisfied with laser treatment. On a visual analogue scale, pruritus and burning improved with a median of 5.5 and 4 points in the full surface ablation group and 3 points on both parameters in the fractional ablation group. Despite the relatively small group of patients, our results suggest that CO_2_ laser ablation improves itching, burning and impact on daily life in tattoo allergy.

## Introduction

Tattooing is a worldwide popular form of body art with an overall prevalence in Europe and the USA of approximately 10–20% [1]. Although it is regarded safe, adverse reactions may occur, including allergic reactions. Many dyes are used for tattooing, but the red dye is most frequently associated with allergic reactions [2, 3]. These reactions are chronic and persistent, characterised by itch, burning and pain and can develop months to many years after getting a tattoo. Regardless of size and location of the affected area, allergic reactions can result in a significantly reduced quality of life [4]. Several clinical subtypes can be recognised of which the ‘plaque type’ is most common. Other, less common types are the ‘excessive hyperkeratotic reaction’ or the ‘ulcero-necrotic reaction’ [5, 6]. Thus far, the responsible allergens have not been identified and the exact patho-mechanism remains unknown [7–9].

Treatment of these allergic reactions is difficult, as tattoo pigments are permanently stored in the dermis. Topical or intralesional corticosteroids are indicated as first-line treatment but effects are often temporary and unsatisfactory [10]. Allopurinol was reported to be effective in one patient; however, symptoms recurred after withdrawal of the drug [11]. Likewise, hydroxychloroquine caused complete regression of pseudolymphomatous tattoo reaction on the trunk and a granulomatous reaction to a red cosmetic tattoo [12, 13]. To achieve permanent remission, the causative allergen needs to be removed. However, the best treatment option to remove the responsible allergen is unknown. Surgical excision, dermatome shaving, Q-switched lasers and ablative CO_2_ lasers are reported as treatment options with permanent results [14, 15]. Nevertheless, each treatment option has its disadvantages, such as possible scarring, infection, risk of generalised allergic reactions and treatment imprecision.

Millán Cayetano et al. considered the continuous wave CO_2_ laser as an effective, safe and precise treatment for improving red tattoo reactions in six patients [16]. Fractional ablation was effectively and safely used for the removal of allergic tattoo reactions in three patients [17, 18]. Apart from these small studies, clinical efficacy has been studied insufficiently. Furthermore, patient-reported outcome measures (PROMs) have not been thoroughly investigated in this field. PROMs are crucial as quality of life and symptom reduction are best assessed by patients themselves [19]. The aim of this study was to report real-life data using ablative laser treatment and to assess PROMs regarding the effectiveness and safety of ablative CO_2_ laser treatment for allergic tattoo reactions.

## Material and methods

We performed a retrospective study in which we included patients with allergic tattoo reactions treated with the 10,600 nm ablative CO_2_ laser (Lumenis Ultrapulse Encore, Lumenis Ltd., Santa Clara, CA, USA) using a handpiece for full surface ablation (2 mm true spot) and/or a handpiece for fractional ablation (DeepFx handpiece, 120 μm beam diameter). Patients were treated between January 2010 and December 2017.

Patients were eligible for inclusion if clinically an allergic reaction was diagnosed and if they were treated with the ablative CO_2_ laser. Allergic tattoo reactions were defined as chronic inflammatory reactions, manifesting in one single colour and persisting for at least 3 months [5, 19]. As there are currently no routine patch tests, the distinction between an allergic tattoo reaction, sarcoidosis and foreign body reaction is mostly clinical (the allergic tattoo reaction is localised to one specific colour) [20, 21]. Furthermore, the histopathology of allergic tattoo reactions is frequently granulomatous as well [22]. Nevertheless, if histologically a granulomatous reaction was observed, laboratory test (including ACE and in some cases sIL-2R) and chest X-ray were performed to exclude sarcoidosis. 

Based on the clinical information provided by the physician, patients and physician made a shared decision about the preferred treatment, either fractional or full surface ablation.

All patients received a test treatment 3 months before the full treatment was started to assess effectiveness and cosmetic outcomes on a small test area.

All patients received infiltration anaesthesia (lidocaine 2% + adrenaline 1:80000).

Fractional ablation settings ranged from 25 to 40 mJ/microbeam and 15–25% density. Full surface ablation was performed with a 2 mm spot, 225 mJ and 10‑30 W combined with wet gauzes to remove carbonised tissue between laser passes. The clinical endpoint of the full surface ablation was the complete removal of red pigments.

After fractional ablation, patients received fucidic acid 20 mg/g cream twice daily for 1 week. After full surface ablation, patients received silver sulfadiazine 10 mg/g cream under hydrofibre absorbent dressings for 1 week. After 1 week when the dressings were removed, patients applied silver sulfadiazine 10 mg/g cream twice daily until healing of the wound was observed. In the case of large areas treated with full surface ablation, systemic antibiotics were used.

Clinical information was obtained from patient’s records and a 25-question questionnaire, addressing 3 topics. The questionnaire was performed retrospectively in December 2017 and January 2018. Overall primary endpoint was patient satisfaction. The questionnaire focused on 3 topics: clinical baseline information, symptoms and outcomes.

### Clinical baseline information

Time interval between placement of tattoo and complaints and, previous treatments. The time interval was based on an ordinal scale. Previous treatment options were given and could be answered with either yes or no. If patients received previous treatment, effects were analysed on an ordinal scale (from no effect to excellent effect).

### Symptoms

Subjective symptoms before and after laser therapy, such as itch and burning. These questions were based on a visual analogue scale (VAS, 0–10), with 0 meaning no itch or burning and 10 meaning the worst itch or burning.

### Outcomes

Overall satisfaction, cosmetic outcome and satisfaction with improvement of pruritus, burning, inflammation and influence on daily life were based on an ordinal scale (from highly unsatisfied to highly satisfied). Evaluation of pain and discomfort during treatment was based on a VAS (0–10), with 0 meaning no pain or discomfort and 10 meaning the worst pain or discomfort. Scar formation and pigment variation could be answered with either yes or no. If present, the burden of scars and pigment variation were based on a VAS (0–10), with 0 meaning no burden and 10 meaning the worst burden.

A statistical analysis was performed. Outcomes between groups were compared using the Student’s *t* test, Mann-Whitney *U* test or Kruskall-Wallis test depending on distribution and the number of groups. Categorical data was compared using the chi2 test or Fisher’s exact test depending on group sizes.

## Results

### Patient characteristics

Sixteen patients (11 women, 5 men) were treated with an ablative CO_2_ laser. Two patients were first treated with the fractional CO_2_ laser without clinical improvement and were therefore also treated with full surface ablation. Therefore, 10 patients were treated with the fractional CO_2_ laser and 8 patients were treated with the full-surface CO_2_ laser.

The median age was 44.5 years. Red or nuances of red made up for 94% of all responsible pigments. Other patient characteristics are shown in Table 1.

**Table 1:** Baseline characteristics of 18 treatments in 16 patients

	All (*n* = 18)	GROUP A (full surface ablation) (*n* = 8)	GROUP B (fractional ablation) (*n* = 10)
Age (years)			
Median [Q1‑Q3]	44.5 [36–52.25]	44.5 [36.25–57.75]	44.5 [35.75‑52.25]
Gender, *n*			
Male	6	5	7
Female	12	3	3
Time between placement of tattoo and complaints, *n*			
<2 weeks	7	2	5
> 2‑ < 4 weeks	3	2	1
> 1‑ < 3 months	3	2	1
> 3‑ < 6 months	4	2	2
>6‑ < 12 months	0	0	0
> 12 months	1	0	1
Histopathological pattern, *n*			
Pseudolymphomatous	4	4	0
Granulomatous	4	2	2
Pseudolymphomatous/granulomatous	1	1	0
Not avalaible	9	1	8
Location, *n*			
Lower leg/ankle	12	6	6
Upper leg	1	0	1
Forearm	3	1	2
Upper arm	2	1	1
Colour, *n*			
Red	17	7	10
Black	1	1	0
Previous treatment, *n*			
Potent topical corticosteroids	6	1	5
Potent topical and intralaesional corticosteroids	7	5	2
Intralesional corticosteroids	3	1	2
Potent topical corticosteroids and other laser treatment	2	1	1

*Q1‑Q3* interquartile range

All patients had received prior treatment, such as topical and/or intralesional corticosteroids and Q-switched laser therapy. Prior treatment either failed or patients desired a more permanent solution.

The number of sessions needed varied between both groups, with a median of 1 in the full-surface CO_2_ laser group and a median of 4 in the fractional CO_2_ laser group (*P* < 001).

### Results from medical files

**Fig. 1:**
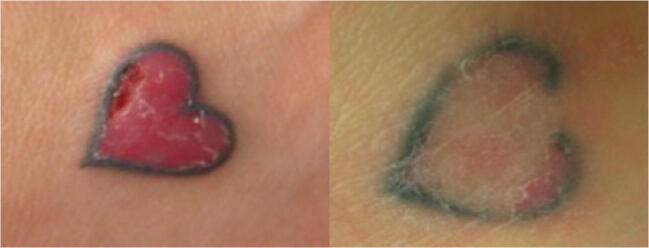
Before and after of a red tattoo reaction treated with fractional ablation

**Fig. 2:**
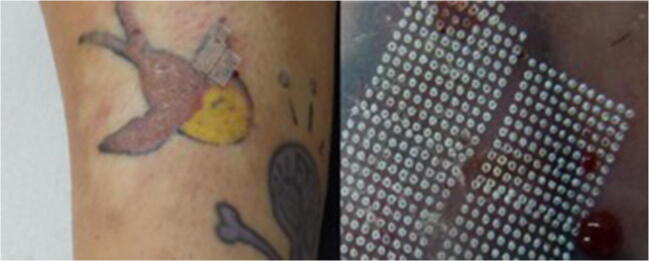
A red tattoo reaction (‘plaque type’) during treatment with fractional ablation

**Fig. 3:**
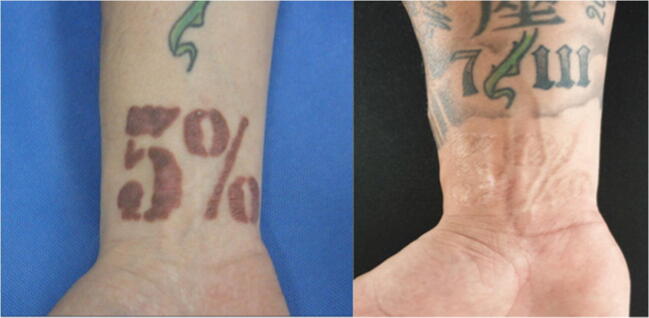
Before and after of a red tattoo reaction (‘plaque type’) on the wrist treated with full surface ablation

The medical files indicated complete remission of symptoms, such as itching, burning or swelling, in six patients (33.3%), which was maintained until the last follow-up. Nine patients had partial remission (50%) and maintained occasional itching or burning. Potent topical corticosteroids were prescribed in some patients to control these residual symptoms. Results of medical files are shown in Table 2 (Figs. 1, 2 and 3).

**Table 2:** Results from medical files, not patient reported

	All (*n* = 18)	GROUP A (full surface ablation) (*n* = 8)	GROUP B (fractional ablation) (*n* = 10)	*P* value
Number of sessions				
Median [Q1‑Q3]	2.5 [1–4.25]	1 [1–1.75]	4 [3–6.25]	< 0.01
Follow-up (months)				
Median [Q1‑Q3]	14 [9.25‑39.75]	8.5 [4.75‑11.75]	31 [20.5–60]	< 0.01
Result after treatment, *n* (%)				1.00
Complete remission	6 (33.3)	3 (37.5)	3 (30)	
Partial remission	9 (50)	4 (50)	5 (50)	
No improvement	3 (16.7)	1 (12.5)	2 (20)

Two adverse events were reported. One patient with skin type 4 developed a keloidal scar after full surface ablation on his upper arm. The other patient developed a generalised allergic reaction 1 day after the fifth fractional CO_2_ laser treatment. Eczema developed around the tattoo, on the arms, face and knees. She was treated with oral antihistamines and topical potent corticosteroids resulting in gradual improvement of the eczematous reaction. This case was reported previously [18].

### Patient-reported outcomes

Two patients did not complete the questionnaire. Thus, 14 patients responded (88%). PROMs are shown in Table 3. From the responding patients, ten patients reported to be either satisfied or highly satisfied with ablative laser treatment (62.5%). Six patients reported to be satisfied with the cosmetic aspect after ablative laser treatment (37.5%), 4 patients were neutral (25%), 6 patients were unsatisfied or highly unsatisfied (37.5%).

**Table 3:** Patient-reported outcome of 16 treated tattoos in 14 patients

	All (*n* = 16)	GROUP A (full surface ablation) (*n* = 8)	GROUP B (fractional ablation) (*n* = 8)	*P* value
Satisfaction with				
Laser treatment, *n* (%)				0.45
Highly satisfied	3 (18.8)	2 (25)	1 (12.5)	
Satisfied	7 (43.8)	4 (50)	3 (37.5)	
Neutral	3 (12.5)	0	3 (37.5)	
Unsatisfied	2 (12.5)	1 (12.5)	1 (12.5)	
Highly unsatisfied	1 (6.3)	1 (12.5)	0	
Cosmetic aspect, *n* (%)				0.59
Highly satisfied	0	0	0	
Satisfied	6 (37.5)	4 (50)	2 (25)	
Neutral	4 (25)	1 (12.5)	3 (37.5)	
Unsatisfied	3 (18.8)	2 (25)	1 (12.5)	
Highly unsatisfied	3 (18.8)	1 (12.5)	2 (25)	
Improvement itch and burning, *n* (%)				0.93
Highly satisfied	4 (25)	3 (37.5)	1 (12.5)	
Satisfied	2 (12.5)	1 (12.5)	1 (12.5)	
Neutral	2 (12.5)	1 (12.5)	1 (12.5)	
Unsatisfied	4 (25)	1 (12.5)	3 (37.5)	
Highly unsatisfied	4 (25)	2 (25)	2 (25)	
Improvement inflammation, *n* (%)				0.52
Highly satisfied	3 (18.8)	2 (25)	1 (12.5)	
Satisfied	6 (37.5)	3 (37.5)	3 (37.5)	
Neutral	2 (12.5)	0	2 (25)	
Unsatisfied	3 (18.8)	1 (12.5)	2 (25)	
Highly unsatisfied	2 (12.5)	2 (25)	0	
Evaluation of laser therapy				
Pain (median [Q1‑Q3])*	3 [1.25–5]	3 [2.25‑4.5]	3.5 [0.25–5]	0.67
Discomfort (median [Q1‑Q3])*	3 [1–5]	2.5 [1.25‑3.75]	3.5 [0.25‑5.75]	0.83
Adverse effects				
Scars, *n* (%)	11 (68.8)	6 (75)	5 (62.5)	1.00
Level of inconvenience (median [Q1‑Q3])*	2.5 [1–6.75]	2.5 [0.75‑7.75]	5 [0.5–7]	1.00
Variation in pigment, *n* (%)	11 (68.8)	7 (87.5)	4 (50)	0.28
Level of inconvenience (median [Q1‑Q3])*	4 [1–5]	4 [0–7]	4.5 [1.75–5]	0.92
Improvement of symptoms				
Itch (median [Q1‑Q3])*	4.5 [7.75‑0.25]	5.5 [8–0]	3 [7.25‑0.25]	0.71
Burning (median [Q1‑Q3])*	4 [7–0]	4 [7–0]	3 [7.75‑0.75]	0.91
Influence on daily life (median [Q1‑Q3])*	2 [4–0]	3 [4–0]	2 [3.75‑0.25]	1.00
Recommendation, *n* (%)	11 (68.8)	7 (87.5)	4 (50)	0.28

Q1‑Q3 = interquartile range

*Registered upon a continuous rating scale from 0 to 10, with 0 being no harm and 10 being extreme harm

Patients rated pain and discomfort of ablative laser therapy with a median VAS score of 3 on both parameters (*P* = 0.67 and 0.83, respectively for pain and discomfort).

Scar formation was reported in 6 patients in the full surface ablation group and 5 patients in the fractional ablation group (*P* = 1.00). Variation in pigment was reported in 7 patients in the full surface ablation group and 4 patients in the fractional ablation group (*P* = 0.28).

Common complaints before starting laser treatment were a burning sensation (93%) and pruritis (100%). Improvement on a VAS scale (0–10) was found, for both, burning sensation and pruritis. When comparing improvement of itch and burning between the full surface ablation group (median of 5.5 for itch and 4 for burning) and fractional ablation group (median of 3 for both itch and burning), it should be noted that more improvement was observed in full surface ablation. However, the difference is not statistically significant (*P* = 0.71 and 0.91, respectively for itch and burning).

The vast majority of the full surface ablation group would recommend this therapy to others (87.5%), in the fractional ablation group 4 patients (50%) gave a recommendation (*P* = 0.28).

## Discussion

Patients suffering from allergic tattoo reactions are burdened with chronic itch and discomfort [4]. Treatment is challenging. Topical or intralesional corticosteroids are convenient options; however, effectiveness varies and is frequently temporary or insufficient. A safe treatment modality with permanent results is needed. Removal of all culprit pigment by surgery or laser ablation is thought to be the best approach. However, there are variable techniques to remove the pigments and little is known about efficacy, side effects and PROMs. In our study, we found that CO_2_ laser therapy can improve the symptoms of allergic tattoo reactions when topical or intralesional corticosteroids are insufficiently effective. Six allergic tattoo reactions showed complete remission (33.3%), 9 showed partial remission (50%) and 3 lesions showed no improvement (16.7%) after a median follow-up of 14 months. Remarkably, in some cases with satisfactory outcomes, we observed some residual red pigment questioning the necessity of complete removal of pigment.

### Fractional and full surface ablation

Patients were overall satisfied with the treatment and reported marked improvement of their symptoms. When comparing our treatment groups, more improvement was reported in the full surface ablation group. Fractional ablation is less invasive with less side effects in comparison to full surface ablation, however, at the cost of multiple treatments and possibly lower efficacy. The difference could not be significantly confirmed. Because of this, the study design and the relatively small group of patients, it cannot be concluded that full surface ablation is superior in effectiveness to fractional ablation.

### Adverse events

It should be noted that adverse effects, such as scarring and allergic reactions, may occur. Full surface ablation has a higher risk of scarring compared to fractional ablation [16]. This could be explained by the fact that conventional CO_2_ lasers ablate the full surface of the skin, whilst fractional CO_2_ lasers ablate a fraction of the skin at a time by emitting microbeams that create microthermal ablation and coagulation zones leaving unaffected tissue around these zones. In our study, no significant difference in scarring between both groups was observed.

In one patient a generalised eczematous allergic reaction was observed after fractional ablation, treated in our Academic Tattoo Clinic. More cases of generalised allergic reactions after fractional laser therapy to treat allergic tattoo reactions have been reported [18, 23]. We assume that full surface ablation completely eliminates pigment-containing cells, thereby preventing systematic uptake. However, in fractional ablation, the ablative channels are small and surrounded by coagulation zones which may be responsible for systemic uptake of allergens [18]. In addition, dyspigmentation is reported several times.

### Other treatment options

Other surgical treatment options are conventional full-thickness excision and dermatome shaving. Conventional excision is only favourable in certain anatomical locations and small size tattoos. In addition, scarring is inevitable. Dermatome shaving is an excellent permanent treatment option. However, in this procedure an experienced plastic surgeon or dermatologist is crucial. Furthermore, in our opinion, CO_2_ lasers have the possibility of treating more accurately in a horizontal plane, resulting in better preservation of the original tattoo design. The pigment-loaded tissue can be removed layer by layer until the desired endpoint, removal of pigment, is achieved. As the depth of the tattoo inks in the skin differs, it is an advantage that depth of laser treatment can be adjusted.

Dermatome shaving may also elicit complications such as scarring, hyper- and hypo-pigmentation, which required additional treatments with intralesional corticosteroids in almost 20% to control scarring [14]. Furthermore, in contrary to dermatome shaving, ablative laser therapy is not a bloody procedure due to surrounding coagulation. Experienced laser surgeons can take advantage of this phenomenon and vary the ratio between ablation and coagulation by adjusting the pulse energy. Therefore, the risk of post-procedure bleeding is smaller.

Another treatment option for allergic tattoo reactions is the Q-switched laser. Unlike ablative lasers, Q-switched lasers are the gold standard for removal of uncomplicated tattoos [24]. Q-switched lasers selectively damage pigment-containing cells, after which pigment particles are released into the systemic circulation. The photomechanical breakdown of pigments may also produce and systemically spread new allergens and harmful chemicals. Several cases of localised, generalised and even anaphylactic allergic reactions have been reported following Q-switched laser tattoo removal in patients with a pre-existent allergic reaction to tattoo pigments or even in prior non-allergic patients [25–28]. Besides the risk of inducing systemic allergic reactions, treatment efficacy for allergic tattoo reactions to red pigment with a Q-switched-laser is compromised because of the limited penetration depth at 532 nm, whilst in ‘plaque reactions’, an evident thickening arises with deeply located pigments. Moreover, Q-switched lasers may require more than ten treatments.

Thus far, this is the largest study of ablative laser therapy in allergic tattoo reactions. Another strength of this study is the long follow-up and the presence of real-life data. Limitations of this study are its retrospective nature, the limited number of included patients and the descriptive analysis. Also, patients were not laser naïve. The response rate of the questionnaire was 88%, which is high. However, it should be noted that some patients had to assess their clinical symptoms years after the initial diagnosis and treatment.

Unfortunately, two patients were lost to follow-up. Furthermore, no validated outcome measures could be used due to a lack of research in this field. Future research should be prospective and include an objective evaluation of improvement of skin inflammation.

Despite the relatively small group of patients, our results suggest that CO_2_ laser ablation (either fractional or full surface ablation) improves itching, burning and impact on daily life in tattoo allergy. It may be implemented as third-line treatment, when topical or intralesional corticosteroids are insufficiently effective. Patients seem to prefer the full surface ablation above fractional ablation, which may result from higher effectiveness and less treatment sessions. However, this could not be statistically confirmed. Patients should be thoroughly informed about the possible risks, especially scar and keloid formation. More evidence is needed before final recommendations can be given.
